# Rapid validation of cancer genes in chimeras derived from established genetically engineered mouse models

**DOI:** 10.1002/bies.201100018

**Published:** 2011-09

**Authors:** Ivo J Huijbers, Paul Krimpenfort, Anton Berns, Jos Jonkers

**Affiliations:** 1) Division of Molecular Genetics, Netherlands Cancer InstituteAmsterdam, The Netherlands; 2) Centre of Biomedical GeneticsAmsterdam, The Netherlands; 3) Academic Medical CenterAmsterdam, The Netherlands; 4) Division of Molecular Biology, Netherlands Cancer InstituteAmsterdam, The Netherlands

**Keywords:** cancer, chimeras, embryonic stem cell, genetically engineered mouse model, non-germline models, recombinase mediated cassette exchange

## Abstract

Recent technological advances have opened the door for the fast and cost-effective generation of genetically engineered mouse models (GEMMs) to study cancer. We describe here a conceptually novel approach for the generation of chimeric GEMMs based on the controlled introduction of various genetic elements in embryonic stem cells (ESCs) that are derived from existing mouse strains with a predisposition for cancer. The isolation of GEMM-derived ESC lines is greatly facilitated by the availability of the newly defined culture media containing inhibitors that effectively preserve ESC pluripotency. The feasibility of the GEMM-ESC approach is discussed in light of current literature and placed into the context of existing models. This approach will allow for fast and flexible validation of candidate cancer genes and drug targets and will result in a repository of GEMM-ESC lines and corresponding vector collections that enable easy distribution and use of preclinical models to the wider scientific community.

## Introduction

Cancer is a disease in which cells display uncontrolled growth accompanied by invasion into normal adjacent tissues. At later stages, some cancers metastasize to other locations in the body via lymph or blood circulation. Studies into the underlying mechanisms have resulted in the identification of a number of key intracellular signaling pathways controlling cell growth and cell death that are often disrupted in tumor cells [1]. Besides these cancer cell-intrinsic pathways external factors also control tumor growth and behavior, e.g. tumor angiogenesis, infiltrating immune cells, and activated mesenchymal cells [2]. For these reasons many elements of cancer biology, including invasion and metastasis, can only be properly studied in the context of intact organisms. Early attempts to model cancer in animals involved subcutaneous or intravenous injection of established human tumor cell lines into immunodeficient mice and monitoring of local tumor outgrowth. Although these xenograft models were effective means to propagate human tumors, they did not model sporadic, de novo tumorigenesis in an immunologically competent host. Over the years mouse models have become much more sophisticated, particularly when genetically engineered mouse models (GEMMs) of cancer entered the arena. Several different types of GEMMs can be distinguished, each with their own benefits and limitations (Table 1). In the more advanced GEMMs, de novo development of “spontaneous” tumors is triggered by spatio-temporally controlled induction of mutations in single cells amidst their natural microenvironment. In this essay, we will not provide an extensive overview of different GEMMs to date, as this has been expertly reviewed by others recently [3, 4]. Instead, we will discuss the development of a new, more flexible GEMM platform designed to meet the increasing demand for swift in vivo validation of potential cancer genes and drug targets identified in functional genomics screens and human cancer genome-sequencing studies [5].

**Table 1:** Comparison between various types of genetically engineered mouse models (GEMMs) of cancer

	Germline GEMMs		Non-germline GEMMs	
	Conventional models	Conditional models	Transplantation Models [11, 43]	Chimeric Models [8, 12]
**Disease**				
Cancer type	Familial	Sporadic	Sporadic	Sporadic
Cancer development is induced by key driver mutations found in human tumors	Yes	Yes	Yes	Yes
Cancer develops in relevant tumor microenvironment	No^a^	Yes	Yes	Yes
Cancer develops in immunocompetent host	Yes	Yes	Yes	Yes
Cancer displays all relevant clinical stages	Usually not	Yes	Yes	Yes
**Model characteristics**				
Rapid cohort generation	No	No	Yes	Yes
Possibility for ex-vivo engineering	No	No	Yes	Yes
Tumor types that can be modeled	Limited by viability of mouse mutants	Many	Limited by availability of stem/progenitor cells	Many
**Investment**				
Lead-time	Long	Long	Moderate	Moderate
Breeding time^b^	Long	Long	None	None
Overall costs^b^	High	High	Moderate	Moderate
**Utility**				
Validation of candidate cancer genes	Moderate	Good, but costly and time-consuming	Good	Good
Validation of drug targets	Moderate	Good, but costly and time-consuming	Good	Good

All tissues have the same genetic rearrangements, including the tumor microenvironment, possibly indirectly affecting tumor behavior.

Estimations for investment in time and relative costs are provided in Figure 4.

## Germline GEMMs: The standard repertoire

GEMMs of human cancers have proven to be valuable models for gaining insight into the different stages of tumorigenesis [3]. Many of the inherited cancer syndromes have been modeled in conventional GEMMs carrying engineered germline mutations in the respective genes. Although conventional GEMMs proved useful to study the in vivo role of cancer genes, their utility for modeling cancer has been limited due to incomplete tumor penetrance coupled with long and highly variable tumor latency.

### Conditional GEMMs

Many of these drawbacks were overcome with the advent of conditional GEMMs [6], which allowed for tissue-specific and time-controlled introduction of conditional mutations using Cre-loxP site-specific recombination. In practice, these mice have been engineered to contain genetic elements flanked by loxP recombination sites. Depending on their configuration, recombination between loxP elements by expressing the Cre recombinase can lead to (mutant) gene activation or inactivation. Tissue-specific Cre expression is achieved by transgenic expression of *Cre* under control of a tissue-specific promoter or by infection of the target tissue with viral vectors containing a Cre recombinase under control of a ubiquitous or cell-type specific promoter. An additional level of control can be achieved by using *CreERT2* transgenes that encode a fusion protein of Cre and the ligand-binding domain of the estrogen receptor (ER). Upon treatment of *CreERT2* transgenic mice with the estrogen analog tamoxifen, the CreERT2 recombinase translocates to the nucleus and catalyzes recombination of loxP elements, allowing for time-controlled activation of conditional mutations.

### Limitations of conditional GEMMs

Conditional GEMMs can recapitulate the stochastic nature of sporadic cancer and often develop one particular tumor type. These models show reduced tumor burden and prolonged lifespan as compared to conventional GEMMs, and are therefore more suitable to study tumor initiation, progression and therapy response [7]. In addition, conditional mouse models can be applied for the validation of candidate cancer genes and drug targets. Unfortunately, a number of logistic and financial barriers prohibit the extensive use of these models in preclinical and translational research. For instance, conditional GEMMs have a long lead-time to be established and validated. The whole process of introducing targeted mutations into embryonic stem cells (ESC) and producing mouse strains from the engineered ESCs is laborious and expensive. Often, multiple conditional mutations are required for development of a specific tumor, thus requiring extensive intercrossing of single mutant mouse strains to produce compound mutant strains of the desired genotype. This process is space- and time-consuming (because it requires complex breeding schemes that involve multiple crosses), inefficient (due to unavoidable generation of offspring with unwanted genotypes), and expensive.

## Non-germline chimeric GEMMs: Modelling without breeding

**Figure 1:**
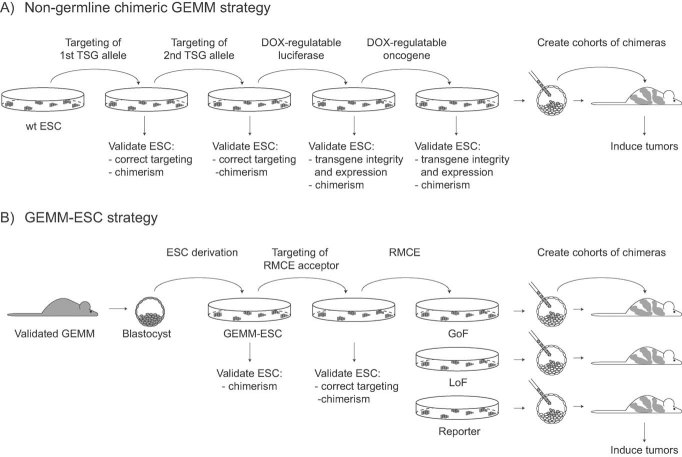
Non-germline chimeric GEMMs. **A:** Current method to obtain non-germline chimeric GEMMs by step-wise modification of wild-type ESCs by introduction of targeted mutations in tumor suppressor genes (TSGs) and random integration of transgenes for doxycycline (DOX) regulatable expression of luciferase reporter and oncogenes [8, 12]. **B:** Outline for GEMM-ESC strategy. ESCs are derived from an existing conditional GEMM containing all the modifications needed to develop a particular type of tumor. The GEMM-derived ESC are validated and targeted with a RMCE acceptor allowing for easy reengineering of the GEMM-ESCs using exchange vectors containing GoF or LoF alleles. Tumors are induced in chimeric animals.

A number of the intrinsic drawbacks of germline GEMMs have been overcome in mosaic mouse models, called non-germline chimeric GEMMs [4, 8]. These models can be subdivided in two groups, transplantation models and chimeric models (Table 1). The transplantation models are generated by implanting somatic stem or progenitor cells into the respective adult tissue of a recipient mouse. These transplanted cells are modified ex vivo to contain tumor-initiating mutations or are derived from tissues of tumor-prone mice. This concept is often used in the hematopoietic system, but has also been applied for tumors from other tissues, such as breast, brain, and liver [9–11]. As this approach is dependent on the isolation of stem/progenitor cells, the range of tumor types that can be modeled is limited by the availability and accessibility of these cells. Chimeric models do not have this limitation, as they are based on genetically engineered ESCs carrying all the essential modifications needed to develop a particular type of cancer. The chimeric mice generated with these ESCs are directly used for tumor induction, allowing for fast cohort generation without tedious intercrosses. The chimeras have a mixture of wild-type cells originating from both host blastocysts and genetically modified cells originating from the cultured ESCs allowing for tumor development in context of normal tissue, recapitulating human tumorigenesis. The downside of this approach is again the long lead-time. The ESCs are modified in a step-wise manner and need to be tested at each individual stage for functionality of the introduced genetic element and also for their contribution to the subsequent chimeras (Fig. 1A) [8, 12]. With these considerations in mind we explored an alternative strategy for generating nongermline chimeric GEMMs that fulfill all requirements for efficient use in translational research. This strategy should meet the following criteria. Firstly, it should yield mouse models that represent the human disease, i.e. de novo tumor development should be driven by key genetic lesions induced in the relevant target tissue in phenotypically wild-type hosts, and display the typical clinical stages of tumor progression in patients.

Secondly, the strategy should allow for rapid ex vivo engineering of ESCs and generation of mouse cohorts, resulting in a flexible platform for side-by-side evaluation of multiple cancer genes or drug targets. Thirdly, the approach should be cost-effective, making it amenable for preclinical and translation research in academic institutions. Finally, the strategy should fulfill some of the three R's for animal-based research (Replace, Reduce and Refine), by reducing the amount of mice required for breeding and by refining GEMMs to improve their utility as preclinical in vivo models of human cancer.

## GEMM-ESC: The fast-track strategy

**Figure 2:**
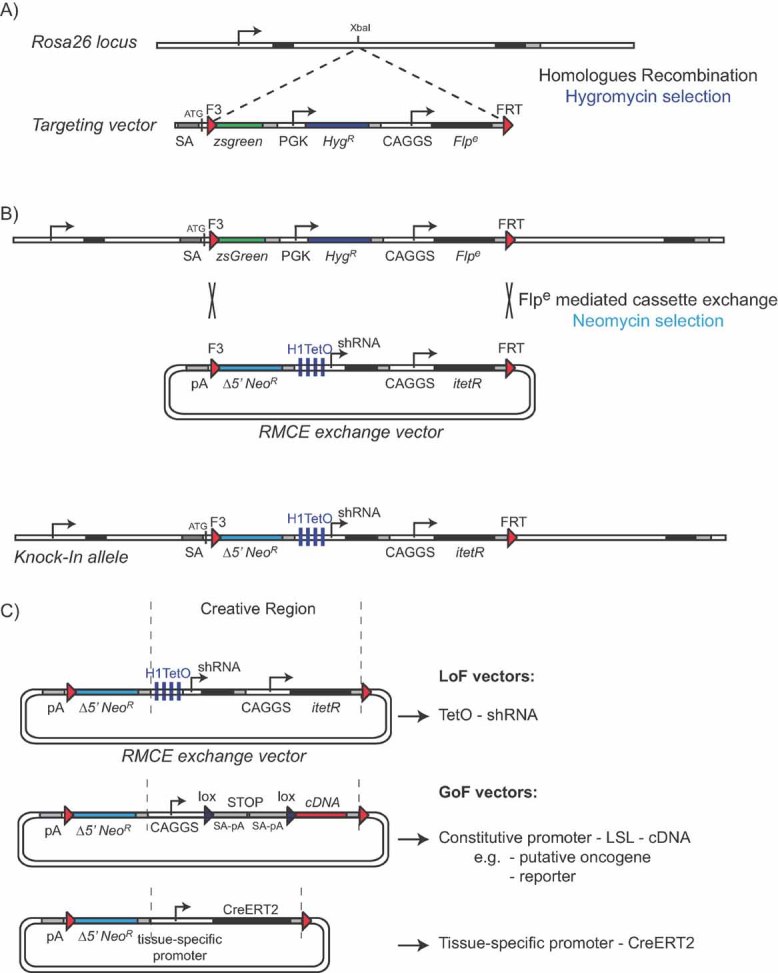
RMCE as described by Seibler and co-workers [36]. **A:** The *Rosa26* allele is targeted with a cassette encoding the ΔATG-zsGreen gene, a Hygromycin resistance (Hyg^R^) marker and the FLPe recombinase. Constitutive PGK and CAGGS promoters control the expression of Hyg^R^ and FLPe, respectively, whereas expression of zsGreen is controlled by the endogenous *Rosa26* gene promoter prior to the F3 recombination site. A FRT recombination site flanks the 3′ end of the targeted locus. **B:** Introduction of doxycycline-inducible shRNA expression constructs via FLPe recombinase-mediated cassette exchange is achieved by co-transfection of the targeted ESCs with a FLPe expression plasmid and an RMCE exchange cassette encoding an F3 site, Δ5′ Neomycin resistance (Neo^R^) marker, the H1-tet promoter, an shRNA of choice, the enhanced tet-repressor itetR controlled by the CAGGS promoter and an FRT site. **C:** The original RMCE exchange vector is ideally suited for LoF vectors. By replacing the region between the dotted lines, named the creative region, these vectors can be adapted to become GoF vectors. Two examples are provided, an inducible cDNA and a tissue-specific CreERT2 gene for spatiotemporal controlled deletion of genes flanked by loxP sites.

As an alternative strategy to produce non-germline chimeric GEMMs, we propose a two-stage approach, named GEMM-ESC, which is outlined in Fig. 1B. In the first stage, ESCs are derived from already existing and validated conditional GEMMs using recently described ESC culture conditions that ensure maintenance of ESC pluripotency (i.e. the capacity of ESCs to contribute to every tissue of the developing embryo) [13]. The GEMM-derived ESC clones are injected into pre-implantation embryos and their contribution to the chimeras is determined. The best performing GEMM-ESC clones are selected and targeted with recombinase mediated cassette exchange (RMCE) acceptors in pre-defined loci [14] (for an example see Fig. 2A).

These RMCE acceptors contain site-specific recombination sites (typically FRT sites) that permit (Flp) recombinase-mediated site-specific integration of custom-designed exchange vectors containing compatible recombination sites (Fig. 2B). These targeted GEMM-ESC clones are again validated for their contribution to chimeric mice. The best performing GEMM-ESC clones are expanded and frozen as a repository for future experiments.

In the second stage, a genetic modification (e.g. an inducible shRNA or a conditional cDNA) is introduced into the RMCE locus of the GEMM-ESC clone of a particular mouse model (Fig. 2C). The GEMM-ESC clone containing the desired modification is used to generate chimeras, in which tumors are induced using the same methods as the ones established for the conditional GEMM from which the ESC clone was derived. The second stage ensures rapid and flexible generation of multiple GEMM-ESC clones with gain-of-function (GoF) or loss-of-function (LoF) alleles for different target genes and allows for side-by-side comparison of the phenotypic effects of these alleles on de novo development and maintenance of sporadic tumors.

### GEMM-ESC versus other non-germline chimeric GEMMs

Our GEMM-ESC approach differs in two aspects from the published non-germline chimeric GEMMs [4, 8]. Firstly, both published models start with wild-type ESC lines that are modified in a step-wise fashion, whereas our GEMM-ESC method starts with ESC lines that already carry all the genetic modifications required for tumor development. This effectively eliminates many of the validation steps required for functional testing of the ESCs at intermittent stages (compare Fig. 1A and B). The GEMM-ESC approach will also reduce the cumulative ESC culture period, thereby reducing the risk of introducing unnoticed mutations.

The second difference between GEMM-ESC and published models is the method used to introduce genetic modifications. In the two published models a classic transgenic approach is applied, which allows for the quick introduction of genetic elements in ESCs. The main limitation of this approach is that it is uncontrolled. Transgenes integrate randomly in the ESC genome possibly affecting expression of endogenous genes at or near the integration site. In addition, random integration of transgenes often occurs at multiple loci aggravating the former problem. Furthermore, the integrity of randomly integrated transgenes is not guaranteed, which is complicated by the fact that transgenes often integrate as concatemers containing varying numbers of transgene copies. Although these issues can be overcome by selection for ESC lines with single or low copy integrations and proper expression of the transgene [8], an approach that starts with a single integration into a predefined expression site is highly preferred. It also has the advantage that it permits for testing of allelic series of a particular gene.

## Stage one: ESC derivation

### Selection of appropriate GEMMs for ESC derivation

In principle, any established GEMM can be used as a starting point for the GEMM-ESC strategy, although not every model will be equally suitable. For instance, some conventional GEMMs are not preferred for ESC derivation because mutations in genes that control cell proliferation or differentiation may affect behavior of ECSs during in vitro culture or production of chimeric mice. Similarly, ESCs derived from mouse mutants with defects in telomere maintenance or DNA damage repair may show increased genomic instability, resulting in accumulation of mutations and genetic variation in individual ESC clones and the ensuing chimeras. Many of these problems can be effectively avoided by focusing on conditional GEMMs, as the conditional knockout alleles function normally. Many conditional GEMMs for human cancers are currently available [3]. In our institute we have characterized and validated conditional GEMMs for breast, lung, brain, and skin tumors [15–21]. In Table 2 we have listed a selection of validated conditional GEMMs that represent good starting points for derivation of ESC lines. A point of caution should be made for conditional GEMMs with a Cre recombinase controlled by a tissue-specific promoter, e.g. K14-*Cre*, WAP-*Cre*, or Pdx-*Cre* (Table 2). Although Cre recombinase is normally not expressed in the germline of these strains, in vitro propagation of ESCs might cause unwanted recombination of conditional alleles due to aberrant Cre expression.

**Table 2:** Validated conditional GEMMs suitable for derivation of ESCs

Tumor type	Conditional alleles	Cre induction	Reference
Hereditary breast cancer	*Brca1*^F/F^;*Trp53*^F/F^	K14-*Cre* or WAP-*Cre*	[18]
Lobular breast cancer	*Cdh1*^F/F^;*Trp53*^F/F^	K14-*Cre*	[15]
Small cell lung cancer	*Rb*^F/F^ ;*Trp53*^F/F^	Intratracheal Adeno-*Cre*	[19]
Non-small cell lung cancer	LSL-*Kras*^G12D^	Intratracheal Adeno-*Cre*	[44]
Mesothelioma	*Nf2*^F/F^;*Trp53*^F/F^;*Ink4a*^-/-^	Intrathoracic Adeno-*Cre*	[17]
Pancreatic cancer	LSL-*Kras*^G12D^ LSL-*Trp53*^R172H^	Pdx-*Cre*	[45, 46]
Melanoma	LSL-*Braf*^V600E^;*Ink4a*^-/-^	Tyrosinase-*CreERT2*	[47]

Therefore, ESCs derived from such GEMMs should always be monitored for recombined alleles before further use. The final chimeric mice obtained from these verified ESC clones will have similar Cre expression in adult tissues as compared to the original model and consequently should have similar tumor characteristics, provided that the ESC clones give rise to good chimeras.

### Isolation of GEMM-derived ESCs

ESCs are derived from the inner cell mass (ICM) of pre-implantation blastocysts [22]. They are typically cultured on feeders of mitotically inactivated fibroblasts in medium containing leukemia inhibitory factor (LIF), which is required for maintenance of a pluripotent state capable of generating any cell type in the body [23]. In the last few years it has become clear that pluripotency of mouse ESCs is governed by three signaling pathways namely the LIF/STAT3 pathway, the fibroblast growth factor (FGF)/ERK pathway and the Wnt pathway [13, 24–27]. These new insights have resulted in the development of defined ESC culture media in which feeder cells and serum have been replaced by small-molecule inhibitors of the FGF4/ERK and Wnt pathways [13]. These new culture media – which are known as 2i or 3i medium depending on the number of inhibitors used – have opened the door for derivation of rat ESCs [28, 29] and ESCs from mouse strains that were previously refractory to ESC derivation, such as the CBA and the MF1 strains [13].

For the GEMM-ESC strategy, we use the new 2i/3i culture conditions to establish authentic ESCs from conditional GEMMs. The feasibility of this approach is exemplified by Nichols and coworkers [30], who used 2i culture medium supplemented with LIF to derive germline-competent ESCs from non-obese diabetes (NOD) mice. These ESC lines had to fulfill a number of criteria. Firstly, ESC lines were verified for having a normal diploid karyotype and showing expression of pluripotency markers Nanog and Oct4. Secondly, their contribution to chimeric mice was validated and the resulting chimeras were evaluated for their ability to give germline transmission. The same parameters were again tested after genetic manipulation of the NOD ESC lines by introducing a DsRED expression cassette via PiggyBac transposition. The DsRED positive NOD ESC clone was indeed able to generate chimeric animals with germline transmission, validating the entire approach. We foresee similar efficiencies in ESC derivation from conditional GEMMs, especially since these models are phenotypically wild-type.

### Validation of chimeric mice

One of the most crucial aspects of the GEMM-ESC strategy will be the validation of the chimeric mice, which should display a high degree of chimerism and develop tumors with similar phenotypes and latencies as the original GEMM. The feasibility of the GEMM-ESC approach is supported by recent reports on the successful generation of chimeric mouse models for breast cancer and non-small cell lung cancer (NSCLC) based on step-wise introduction of multiple genetic modifications into wildtype ESCs [8, 12]. Chimeric mice derived from *Ink4a/Arf*^*-/-*^ ESCs carrying transgenic constructs for lung-specific, doxycycline-inducible expression of mutant HER2^V659E^ developed doxycycline-inducible lung tumors, which regressed upon doxycycline withdrawal [8]. The tumor burden in these chimeras correlated with the degree of coat-color chimerism. Longer tumor latency was observed in the chimeric mice compared to the conventional mouse mutants, possibly reflecting reduced numbers of premalignant lesions in chimeras due to the presence of both wild-type and genetically modified cells. If tumor penetrance in chimeric mice is too low, despite high degree of coat color chimerism and effective induction of oncogenic mutations, completely GEMM-ESC derived F0 mice can be produced by aggregation of GEMM-ESCs with tetraploid mouse embryos [31, 32]. Alternatively, chimeric mice can be bred with the original GEMM to produce GEMM-ESC derived F1 mice. An advantage of the latter approach is that it will generate experimental F1 mice as well as control mice lacking the additional mutation that was introduced in the GEMM-ESCs.

## Stage two: Genetic modification of GEMM-derived ESCs

### Gene targeting in GEMM-derived ESCs

Gene targeting in GEMM-derived ESC lines may be complicated by two factors. One, existing targeting vectors often have homology arms derived from 129 or C57BL/6 genomic DNA, as the commonly used mouse ESC lines are derived from these two inbred strains. However GEMMs have often been backcrossed to other backgrounds such as FVB/n or BALB/c. Using the original, non-isogenic targeting constructs will likely result in reduced gene targeting efficiencies due to the presence of DNA polymorphisms between the ESC genome and the targeting vector [33]. Therefore, existing targeting vectors may need to be retrofitted with homology arms isogenic to the appropriate background. In practice, this means that the strain background of the target locus in the derived ESCs is determined by screening for nearby polymorphisms. Upon identification of the strain background a suitable BAC clone is identified and used for further manipulations. Alternatively, custom designed homology arms are amplified by long-range PCR with a high-fidelity polymerase, using DNA isolated from the GEMM-ESC as template.

The second aspect that may hamper efficient genetic modification of GEMM-derived ESCs is the fact that gene targeting by homologous recombination has not yet been tested in mouse ESC lines derived in 2i/3i culture medium. Nonetheless, we do not expect extensive difficulties as it has recently been shown that a rat ESC line derived in 3i culture medium permits targeting of the *Trp53* gene albeit with a low efficiency of 1–4% [34]. Of note, only a single targeting event in the GEMM-derived ESCs is required to introduce the recombinase-mediated cassette exchange (RMCE) vector. All subsequent manipulations are independent of homologous recombination (Fig. 2).

### Re-engineering of GEMM-ESCs by recombinase mediated cassette exchange

Recombination-mediated cassette exchange is a technique that allows for efficient and scalable engineering of the mouse genome [35]. This strategy involves the exchange of a genomic region flanked by heterotypic recombination sites (e.g. wild-type and mutant FRT sites) with an incoming region that is flanked by the same two recombination sites (Fig. 2A, B). In cultured ESCs, efficient selection of clones with the correct recombination event is assured by a switch in antibiotic resistance.

For instance, Seibler and coworkers used an RMCE system in which a hygromycin resistance marker flanked by wild-type and mutant FRT recombination sites was replaced by a neomycin resistance gene flanked by the same recombination sites following expression of Flp recombinase [36] (Fig. 2A, B). In addition to the neomycin resistance gene a doxycycline-inducible shRNA expression cassette was also introduced.

A major benefit of this approach is that a single intact copy integration is achieved into a predefined genomic region, effectively creating a knock-in allele. Furthermore, RMCE requires only a single targeting of ESC clones with the acceptor cassette, in general a slow and laborious process. All subsequent re-engineering steps are achieved by simple co-transfection of ESCs with a custom-designed RMCE vector and also the Flp expression vector. In principle any locus can be used for recombinase-mediated targeting. Two loci have been extensively used, i.e. *Rosa26* and *ColA1* [36, 37]. Both loci have been shown to allow for ubiquitous expression of the transgene.

### Introduction of designer alleles

**Figure 3:**
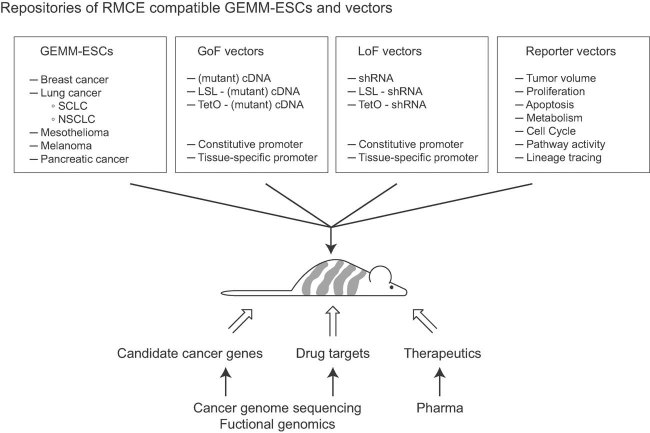
Generation of repositories of RMCE-compatible ESCs from various GEMMs and collections of GoF and LoF vectors enables rapid in vivo validation of candidate cancer genes and drug targets identified in functional genomics screens or cancer genome sequencing projects [5]. Collections of RMCE-compatible reporter vectors permit in vivo monitoring of tumor-related processes via non-invasive imaging techniques.

The flexibility to introduce different genetic elements in the RMCE locus is very high (Figs. 2C and 3). GoF alleles can easily be introduced by placing a cDNA of interest under control of a constitutive or tissue-specific promoter. An additional level of control can be obtained by placing a Lox-STOP-Lox (LSL) cassette containing a transcriptional terminator between the promoter and the cDNA, which will ensure expression of the cDNA only in cells expressing functional Cre recombinase. Attractive cDNAs are for instance putative oncogenes, mutant forms of known oncogenes and *CreERT2* constructs for tamoxifen-inducible tissue-specific recombinase expression.

RMCE also permits rapid introduction of various reporter constructs for non-invasive and longitudinal monitoring of tumor volume, for tumor lineage tracing and for molecular imaging of cellular processes such as metabolic activity, proliferation, apoptosis, and the activity of specific signaling pathways. Introduction of reliable reporters for tumor growth will be of immediate interest, as many conditional GEMMs for internal cancer types would benefit from improved tumor monitoring. Such reporters may include codon-optimized firefly luciferase for non-invasive imaging or fluorescent proteins for intravital imaging of tumor cells using two photons or multiphoton microscopy [38, 39]. The flexibility for side-by-side comparison of different fluorochromes or luminescence markers in specific tumors in chimeric mice will facilitate identification of the most optimal reporter for each individual GEMM-ESC tumor model.

LoF alleles can also be introduced by RMCE, by employing constructs for constitutive or tissue-specific expression of shRNA. An additional level of control can be achieved by using the Tet-ON or Tet-OFF system for doxycycline- regulatable expression of shRNAs. An RMCE cassette is available for doxycycline-regulatable shRNA expression in the *Rosa26* locus [36] (Fig. 2A, B). The utility of inducible shRNA expression for in vivo drug target validation was demonstrated by Xue and co-workers, who used a doxycycline-regulatable shRNA against *Trp53* to establish a chimeric mouse model of liver cancer [40]. Discontinuing shRNA expression by doxycycline administration to mice with established liver tumors caused P53 reactivation and concomitant tumor regression.

### Potential drawbacks of the GEMM-ESC approach

We foresee that the use of RMCE in GEMM-ESCs will create a flexible and rapid in vivo platform for side-by-side comparison of potential cancer genes and drug targets. Nevertheless, we also foresee potential drawbacks of the GEMM-ESC approach. One limitation is that it can only be used for tumor intrinsic modifications, since the study of tumor extrinsic elements is difficult to model in a non-germline chimeric setting as wild-type stromal cells will likely compensate for the effects of the modified allele in for instance mesenchymal or endothelial cells.

A second drawback for the chimeric GEMM-ESC models is that they will not always be directly comparable to the original model in terms of tumor incidence and latency. How they compare needs to be determined on a model-to-model basis. We anticipate that comparable tumor latencies will be observed for models with single, clonal tumors, whereas models with multiple, oligoclonal tumors may yield GEMM- ESC chimeras with prolonged latencies due to lower tumor burden. On the flip side the GEMM-ESC approach may allow for improved side-by-side comparison of cancer genes due to elimination of genetic background differences and associated phenotypic variation arising from modifier loci introduced via intercrosses.

Another aspect of the GEMM-ESC approach that needs to be assessed is the correlation between the observed coat color chimerism and the real chimerism in the tissue of interest. Experimentally, a ratio can be established by introducing a fluorescent marker in the ESCs to determine the ratio of fluorescent versus non-fluorescent cells in the target tissue in chimeric mice. This approach will allow the determination of a cut-off point for the use of chimeras. Ideally, the GEMM-ESC approach would use F0 mice that are fully derived from ESCs. Several injection techniques have been established to achieve this goal [31, 32, 41].

A final limitation for the general use of the GEMM-ESC approach is the dependence on specialized facilities to produce the F0 chimeras or ESC derived mice. Many institutes have core facilities for mouse blastocysts injections, and the injection of GEMM-ESC clones is no different from the injection of wild-type ESC clones. Only the injection capacity needs to be increased to meet the demand.

## Conclusions

**Figure 4:**
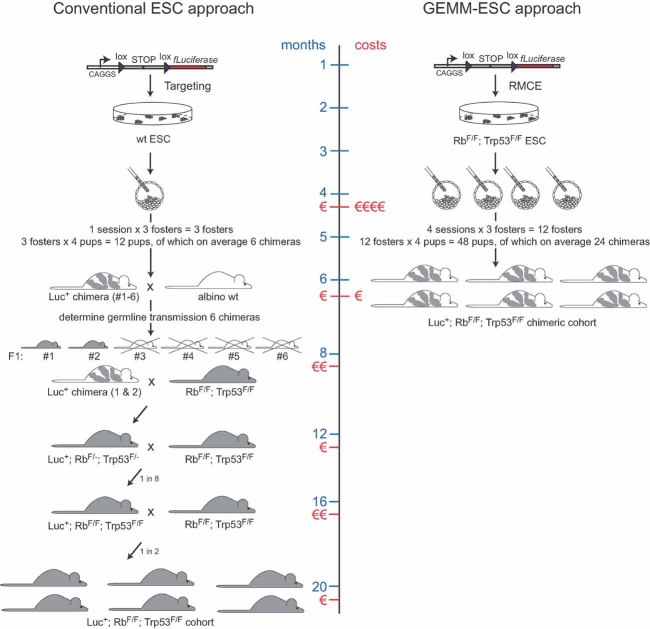
Side-by-side comparison for investment in time and costs between conventional targeting in wild-type ESCs and the GEMM-ESC approach. A typical example is given for a tumor model with four modified alleles, i.e. *Rb^F/F^*;*Trp53^F/F^*, in which one additional allele is introduced, a luciferase reporter. A conditional luciferase expression construct is introduced in wild-type ESCs or *Rb^F/F^*;*Trp53^F/F^* ESCs via either classic targeting or RMCE, respectively. Both approaches require blastocyst injections, though the GEMM-ESCs require more injections to directly establish a cohort of F0 chimeras. For the conventional wild-type ESC approach the number of crosses is shown to establish a similar cohort of compound mutant mice of the desired genotype. The ratio of mice with the right genotype generated from each cross is indicated. Note that the GEMM-ESC approach will deliver a considerable gain in time to establish a cohort (approximately 14 months), and a reduction in costs as the initial higher costs for chimera production are gained back by the absence of any breeding.

The power of the GEMM-ESC approach lies in the fact that it starts from well-established GEMMs, taking advantage of the time that has already been invested in characterizing, validating and optimizing these models. Generation of GEMM-derived ESCs will create a cumulative repository that permits easy distribution of these preclinical models to the wider scientific community and provides an alternative for cryopreservation of mouse strains by embryo or sperm freezing (Fig. 3). Should the GEMM-ESC approach live up to its promise it will revolutionize the way we work with GEMMs. Currently, a large proportion of genetically engineered mice in animal facilities are kept for breeding purposes to maintain the various strains and only a minority is used in experiments (Fig. 4). The GEMM-ESC approach will result in a substantial reduction of breeding colonies over time. Initially, breeding pairs of a specific GEMM are required for derivation of ESCs, but once this is accomplished the emphasis will shift towards generating chimeric animals from GEMM-ESCs carrying various additional modifications. These chimeric mice will be directly used for in vivo experiments. In addition, the GEMM-ESC approach will facilitate the use of complex GEMMs in cancer research as the introduction of additional modifications is greatly simplified due to the RMCE approach in GEMM-derived ESCs. The researcher only needs to customize the exchange vector and introduce this in the ESCs (Fig. 2C and 3).The modified ESCs are subsequently used for the production of chimeric mice by injection into pre-implantation embryos. The next few years will be crucial to assess whether the GEMM-ESC strategy will be a valid alternative to the current approaches to model cancer in GEMMs. The first proof-of-concept of the GEMM-ESC approach has recently been published [42].

## Abbreviations

ESC: embryonic stem cell
GEMM: genetically engineered mouse model
GoF: gain-of-function
LoF: loss-of-function
LSL: Lox-STOP-Lox
RMCE: recombinase mediated cassette exchange.
